# Safety and efficacy of CD22 and CD19 CAR-T bridging auto-HSCT as consolidation therapy for AYA and adult B-ALL

**DOI:** 10.1038/s41408-023-00837-3

**Published:** 2023-05-03

**Authors:** Yan Qiu, Chao-Ling Wan, Ming-Zhu Xu, Hai-Xia Zhou, Mei-Jing Liu, Wen-Jie Gong, Li-Qing Kang, Ai-Ning Sun, Lei Yu, De-Pei Wu, Chong-Sheng Qian, Sheng-Li Xue

**Affiliations:** 1 National Clinical Research Center for Hematologic Diseases, Jiangsu Institute of Hematology, The First Affiliated Hospital of Soochow University, Suzhou, People’s Republic of China; 2 Institute of Blood and Marrow Transplantation, Collaborative Innovation Center of Hematology, Soochow University, 215000 Suzhou, People’s Republic of China; 3 Shanghai Unicar-Therapy Bio-medicine Technology Co., Ltd, Shanghai, People’s Republic of China; 4 Institute of Biomedical Engineering and Technology, Shanghai Engineering Research Center of Molecular Therapeutics and New Drug Development, School of Chemistry and Molecular Engineering, East China Normal University, Shanghai, People’s Republic of China

**Keywords:** Cancer immunotherapy, Acute lymphocytic leukaemia

A remarkable complete remission (CR) rate has been achieved with chimeric antigen receptor-modified T (CAR-T)-cell treatment in patients with recurrent or refractory acute B-lymphocytic leukemia (r/r B-ALL) [1, 2]. However, recurrence has been reported in 30%-70% of r/r B-ALL patients [3, 4]. Multiantigen CAR-T cells and the combination of CAR-T cells with other regimens may reduce the relapse rate and prolong leukemia-free survival (LFS) and overall survival (OS) [5]. Combining CAR-T cells with allogeneic hematopoietic stem cell transplantation (allo-HSCT) may be a promising strategy; however, the benefit of CAR-T-cell therapy bridging allo-HSCT remains controversial [6]. Allo-HSCT after CAR-T-cell therapy has been shown to reduce relapse, while nonrelapse mortality (NRM) offsets the benefit, resulting in no significant difference in event-free survival (EFS) and OS outcomes [7, 8].

An analysis by the Acute Leukemia Working Party of the EBMT showed that although the recurrence rate associated with autologous stem cell transplantation (auto-HSCT) was higher, there was no significant difference in LFS or OS outcomes due to the lower NRM rate in auto-HSCT recipients [9]. A retrospective analysis from the European Working Group on ALL in Adults (EWALL) demonstrated that the 5-year LFS rate was significantly higher in the group with lower minimal residual disease (MRD) before auto-HSCT (<10^–3^) than in the group with higher MRD (>10^–3^). This finding indicated that patients with a deep remission status before auto-HSCT benefit from the treatment [10].

A phase I/II clinical trial (NCT05470777) was conducted to assess the safety and efficiency of CD22 and CD19 CAR-T cells bridging auto-HSCT in adolescent and young adult (AYA) and adult patients with B-ALL at our center. The main purpose of this study was to reduce relapse and NRM by this strategy, which may fully exploit the advantages of CAR-T-cell therapy and auto-HSCT and ultimately improve LFS and OS outcomes.

All patients received standard induction and consolidation chemotherapy. Two Ph-positive patients received tyrosine kinase inhibitors (TKIs) during induction and consolidation therapy. Peripheral blood mononuclear cells (PBMCs) were collected at week 4 after induction chemotherapy. Autologous stem cell steady-state mobilization and collection were performed 6–8 weeks after sequential infusion of CD22^+^ and CD19^+^ CAR-T cells (4-1BB as a costimulatory molecule). After conditioning with modified BuCy, auto-HSCT was conducted. Patients were followed up to monitor MRD by flow cytometry in all patients and IgH rearrangement (with a sensitivity of 10^–6^) assessed by second-generation gene sequencing in four patients (Fig. 1a). Patients with MRD > 10^–3^ (by flow cytometry) after CAR-T-cell treatment were withdrawn from the study and received allo-HSCT. Specific details of the study protocol can be found in the methods section of the supplementary materials and Supplementary Fig. 1.

**Fig. 1:**
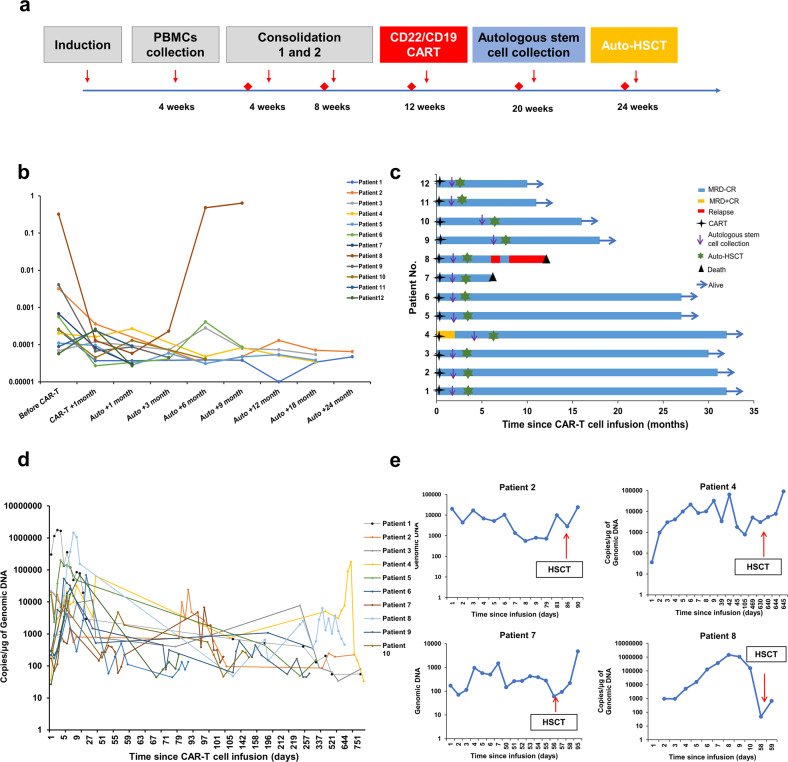
Study design, MRD by flow cytometry, long-term prognosis, and expansion of CAR-T cells. **a** Protocol of the clinical trial. The red diamond icon represents bone marrow puncture. **b** The graph shows a trend of decreasing MRD in patients after CD22/CD19 CAR-T therapy. Except one patient who experienced relapse, MRD remained at a low level (10^-4^) in all patients during follow-up. **c** The graph shows the leukemia status from the start of CAR-T infusion (d0). The blue bar indicates MRD-negative CR. The yellow bar indicates MRD-positive CR. The red bar indicates relapse. Two patients died during follow-up. **d** The copies of CAR-T cells in the peripheral blood were measured by qRT‒PCR after infusion. All the patients’ CAR-T-cell copies were still detectable in the peripheral blood and could be detected in 6 patients for more than 2 years. **e** We also detected CAR-T cells after stem cell infusion in 4 patients and found that CAR-T copies were elevated in all patients.

A total of 12 patients were enrolled in this study. Baseline characteristics are shown in Supplementary Table 1. Two (15.4%) patients developed grade 1–2 cytokine release syndrome (CRS), and 11 (84.6%) patients did not exhibit CRS (Table 1). No immune effector cell-associated neurotoxicity syndrome (ICANS) was observed. One patient died from a pulmonary infection 2 months after auto-HSCT. The median times from CAR-T therapy to recovery of neutrophils, platelets, and hemoglobin were 5.5 (range, 4–16) days, 7 (range, 4–20) days, and 29.5 (range, 10–55) days after supportive treatment, respectively. All patients developed B-cell aplasia. The median time to CD19-positive cell recovery was 7 (range, 4–12) months. After auto-HSCT, all the patients recovered from granulocyte deficiency after 10 (range, 9–12) days and were free from platelet transfusion after 11 (range, 8–14) days.

**Table 1:** Toxicities and outcomes.

	Ph^-^ B-ALL	Ph^+^ B-ALL	Ph-like B-ALL	Total
Number of patients, *n* (%)	9(75)	2(16.7)	1(8.3)	12(100)
*Incidence of CRS according to the grade, n (%)*				
0	7(70)	2(20)	1(10)	10(83.4)
1	1(100)	0	0	1(8.3)
2	1(100)	0	0	1(8.3)
*Incidence of ICANS according to the grade, n (%)*				
0	9(75)	2(16.7)	1(8.3)	12(100)
*Response after chemotherapy, n (%)*				
MRD^-^ CR	6(66.7)	2(22.2)	1(11.1)	9(75)
MRD^+^ CR	2(100)	0	0	2(16.7)
NR	1(100)	0	0	1(8.3)
*Response after CAR-T infusion, n (%)*				
MRD^-^ CR	9(75)	2(16.7)	1(8.3)	12(100)
*Response after auto-HSCT, n (%)*				
MRD^-^ CR	8(72.7)	2(18.2)	1(9.1)	11(91.7)
Relapse	1(100)	0	0	1(8.3)

*Ph-B-ALL* Ph-negative B-lymphocytic leukemia, *Ph+B-ALL* Ph-positive B-lymphocytic leukemia, *Ph-like B-ALL* Ph-like B-lymphocytic leukemia, *CRS* cytokine release syndrome, *ICANS* immune effector cell-associated neurotoxicity syndrome, *MRD* minimal residual disease, *CR* complete remission, *NR* no remission, *auto-HSCT* autologous stem cell transplantation.

Before CAR-T-cell infusion, 9 (75%) patients achieved MRD-negative CR, 2 (16.7%) patients achieved MRD-positive CR, and 1 (8.3%) patient presented with no remission (NR). The patient who achieved NR was not treated with bridging chemotherapy before CAR-T-cell therapy. After CAR-T therapy, all patients achieved MRD-negative CR (Table 1 and Supplementary Fig. 1). Patients showed a decreasing trend in MRD monitored by flow cytometry after CAR-T therapy (Fig. 1b). In addition, four patients’ IgH rearrangements were detected before and after CAR-T therapy simultaneously. These four patients also achieved negative IgH rearrangement after CAR-T therapy. At the last follow-up on December 1st, 2022, the median follow-up time for 12 patients was 24 months (range, 5–32 months). There were 83.3% of enrolled patients who maintained MRD-negative CR. A continuous MRD-negative CR was observed in 6 patients for more than 2 years and in 3 patients for more than 1 year. The median OS and LFS outcomes in all 12 patients were not reached. One (8.3%) patient experienced relapse. This patient developed CD22 and CD19 antigen-negative relapse 7 months after auto-HSCT. The patient has ongoing CAR persistence at relapse. She then underwent allo-HSCT but died from progressive disease 2 months after allo-HSCT (Fig. 1c). Subgroup analysis was used to analyze the factors affecting the prognosis of patients. Age, white blood cell (WBC) count at initial diagnosis and cytogenetic risk group were incorporated (Supplementary Fig. 3).

CAR-T cells persisted in all patients during follow-up (range, 5–32 months), including 6 patients beyond 2 years and 4 patients beyond 1 year (Fig. 1d). We assessed CAR-T cells in the stem cell apheresis fraction in 3 patients. The existence of CAR-T cells was found in autologous stem cells. We also detected CAR-T cells in the peripheral blood after stem cell infusion in 4 patients, and CAR-T copies were elevated in all patients (Fig. 1e).

Relapses after CAR-T-cell therapy have become a key challenging issue to address. We adopted sequential infusion of CD22 and CD19 CAR-T cells. CD19/CD22 “cocktail” CAR-T-cell treatments were reported for r/r B-ALL, and 96% of patients achieved CR with LFS durations of 3–11 months and a median LFS time of 13.6 months. However, relapse occurs in approximately 40% of patients [11]. Another way to prevent relapse is the combination of CAR-T and allo-HSCT. In a study of CD19 CAR-T for r/r Ph-positive B-ALL, 30/51 CR patients received consolidation allo-HSCT, and those who received allo-HSCT had better 2-year OS and 2-year EFS outcomes than those who did not [12]. However, due to complications such as graft-versus-host disease (GVHD) and NRM, some studies have shown that consolidation allo-HSCT does not prolong OS times. In an open-label pragmatic clinical trial, among 21 patients who received consolidative allo-HSCT, a significant difference was found in EFS and RFS outcomes, and no difference was found in the OS outcome [13]. Whether patients benefit from CAR-T-cell bridging allo-HSCT still needs further confirmation.

The weak graft-versus-leukemia (GVL) effect of autologous stem cells and the possible presence of leukemia cells may lead to a high relapse rate after auto-HSCT. A retrospective study showed that compared to MSD-HSCT and URD-HSCT, there was no significant difference in 2-year OS and 2-year LFS outcomes due to a considerably lower 2-year NRM rate than in the other two groups, despite a significantly higher 2-year relapse rate with auto-HSCT [9]. In addition, a retrospective analysis showed that deep remission status before auto-HSCT was effective in extending the 5-year LFS rate and preventing relapse [14]. In our research, MRD was monitored by flow cytometry before and after CAR-T-cell therapy. We found that patients’ MRD showed a decreasing trend, which suggested that CD22/CD19 CAR-T cells could lead to deeper remission. With a sensitivity of 10^-6^, four patients’ IgH rearrangements were detected after CAR-T therapy, and all patients achieved negative IgH rearrangement after CAR-T therapy. As mentioned before, a lower MRD before auto-HSCT could significantly prolong the LFS duration. We used CAR-T cells to achieve deeper remission before auto-HSCT, and this new strategy may lead to LFS and OS outcome benefits.

Although patients received a myeloablative conditioning regimen (BUCY) prior to transplantation, CAR-T-cell expansion was detected after auto-HSCT, and CAR-T cells persisted during the long-term follow-up. Two patients had a small increase in MRD 6 months after auto-HSCT, but MRD declined rapidly concomitant with an increase in CAR-T-cell copies. Interestingly, we detected CAR-T cells in the autologous stem cell apheresis fraction of 3 patients by qRT‒PCR, although the proportion was low. The autologous stem cells collected after CAR-T therapy may result in the presence of CAR-T cells among autologous stem cells. The increase in CAR-T-cell copies after auto-HSCT and the long persistence of CAR-T cells may be due to the infusion of CAR-T cells mixed with stem cells. The second CD22 and CD19 CAR-T cells were sequentially infused in the later stage of research (NCT05470777). Moreover, the myeloablative conditioning regimen (BUCY) played a role as a lymphodepletion regimen for CAR-T cells mixed with stem cells or the second CAR-T cells in our study. The BUCY regimen may create a favorable immune environment for CAR-T cells, which could improve their expansion, persistence and clinical activity while reducing the potential for anti-CAR immune responses [15]. Related experiments are in progress.

In conclusion, our preliminary study demonstrated that autologous CD22 and CD19 CAR-T-cell infusion bridging auto-HSCT as a consolidation strategy showed favorable safety and efficacy in B-ALL. CD22 and CD19 CAR-T cells induced a deeper remission prior to auto-HSCT and may allow patients to benefit from both OS and LFS.

## Supplementary information

Supplemental material

## Data Availability

The data generated in this study are available upon request from the corresponding author.
