# Supplementary material for: Safety and efficacy of CD22 and CD19 CAR-T bridging auto-HSCT as consolidation therapy for AYA and adult B-ALL
Source: Blood Cancer J. 2023 May 3;13(1):66. doi: 10.1038/s41408-023-00837-3 (PMC10156719; doi:10.1038/s41408-023-00837-3)
Supplement: Supplementary file 1 — Supplemental material [file 41408_2023_837_MOESM1_ESM.docx]

**Materials and methods**

**Inclusion and exclusion criteria.**

All patients were enrolled according to this trial's inclusion and exclusion criteria. Eligible subjects met all of the following criteria: (1) primary diagnosis of B-ALL with (a) no suitable allogeneic HSCT donor or (b) refusal of allogeneic HSCT; (2) positive expression of CD19 and CD22 on blasts in the peripheral blood or bone marrow; (3) cardiac ultrasound left ventricular ejection fraction ≥50%; creatinine ≤1.6 mg/dl; alanine transaminase (ALT) and aspartate aminotransferase (AST) ≤3 times the normal range and total bilirubin ≤2.0 mg/dl; pulmonary function ≤ grade 1 dyspnea (CTCAE v5.0) with oxygen saturation > 91% without oxygenation; (4) aged 15-65 years (including 15 and 65 years), regardless of sex; (5) T-cell amplification test pass; and (6) expected survival > 3 months.

The exclusion criteria were as follows: (1) patients with extramedullary lesions; (2) a combination of other malignant tumors; (3) patients previously treated with anti-CD19 and/or CD22 and/or CD3 therapies; (4) immunosuppressant use within 2 weeks before signing informed consent or plan to use immunosuppressants after signing informed consent; (5) uncontrolled active infections; (6) HIV infection; (7) active hepatitis B or hepatitis C infection; (8) history of severe tachyphylaxis to aminoglycoside antibiotics; and (9) history or presence of clinically relevant central nervous system (CNS) pathology, such as epilepsy, generalized seizure disorder, paresis, aphasia, stroke, severe brain injuries, dementia, Parkinson's disease, cerebellar disease, organic brain syndrome, or psychosis.

**Ethics statement.**

The study protocol was approved by the Ethics Committee of the First Affiliated Hospital of Soochow University. Written informed consent was obtained from all patients.

**Manufacturing of CD22 and CD19 CAR-T cells.**

PBMCs were obtained from the enrolled patients by leukapheresis. T cells were separated using anti-CD3 magnetic beads (Miltenyi, Biotec, Bergisch-Gladbach, Germany), stimulated with anti-CD3/CD28 monoclonal antibodies (Miltenyi, Biotec, Bergisch-Gladbach, Germany) and transduced with recombinant lentiviral vectors encoding the CD19 and CD22-BB-z transgenes. CD22 and CD19 CAR-T cells were cultured in AIM-V media (Gibco, NY, USA) supplemented with 10% autologous human serum, 100 IU/mL recombinant human IL-2 (PeproTech, Rocky Hill, USA), 5 ng/mL recombinant human IL-7 (PeproTech) and 5 ng/mL recombinant human IL-15 (PeproTech) for 12-14 days before infusion. The quality of CAR-T cells was checked throughout the manufacturing process.

**Induction and consolidation chemotherapy.**

Induction therapy consisted of vindesine (4 mg/d intravenously every week on days 1, 8, 15, 22), idarubicin (6 mg/m2/d intravenously every week on days 1, 8, 15, 22), and dexamethasone (10 mg/m2/d intravenously daily on days 1-21, tapered on days 22-28).

Consolidation therapy consisted of two courses of systemic chemotherapies, one cycle of medium-dose cytarabine (2 g/m2/d intravenously q12h on days 1-2) concurrent with pegaspargase (2500 IU/m2 IM on day 3) and one cycle of high-dose methotrexate (3 g/m2/d intravenously on day 1) concurrent with pegaspargase (2500 IU/m2 IM on day 3) (Supplementary Fig. 1).

**Preparative lymphodepletion chemotherapy and CD22/CD19 CAR-T infusion.**

All patients received the FC lymphodepletion regimen (fludarabine 30 mg/m^2^; cyclophosphamide 0.3 g/m^2^). Three days later, separate doses of 5×10^6^/kg CD22 CAR-T and CD19 CAR-T cells were sequentially infused on day 1 (40% CD22 CAR-T), day 2 (60% CD22 CAR-T), day 3 (40% CD19 CAR-T) and day 4 (60% CD19 CAR-T) (Supplementary Fig. 2). The patients’ temperature, blood pressure, heart rate, respiration and blood oxygen were monitored before, during, and after infusion.

**Adverse event and outcome measurements.**

Adverse events were evaluated with CTCAE V5.0 from the date of entry into the trial. Cytokine release syndrome (CRS) and immune effector cell-associated neurotoxicity syndrome (ICANS) were graded based on the NCCN guidelines^1^. Severe CRS and ICANS were defined as grade ≥3. Efficacy was assessed on day 30 after chemotherapy, day 30 after CAR-T-cell therapy and day 30 after auto-HSCT. CR was defined as < 5% bone marrow blasts. Flow cytometry was used to detect MRD in all patients. Four of these patients also had MRD detected by IgH rearrangement. MRD-negative CR was defined as CR with MRD<1*10^-4^ in the bone marrow assessed by flow cytometry. The 2-year LFS and OS rates were calculated to assess long-term patient outcomes. LFS was measured from the date of achievement of remission from CAR-T therapy until the date of relapse from CR or death from any cause. OS was measured from the date of CAR-T-cell therapy to the date of death from any cause.

**Detection of CAR-T-cell copies.**

After CAR-T-cell infusion, the expansion and persistence of CAR-T cells were monitored by real-time quantitative polymerase chain reaction (qRT‒PCR) and reported as transgene copies per µg of genomic DNA. DNA was extracted from the patient’s peripheral blood sample via the TIANamp Blood DNA Kit (TIANGEN, China) according to the manufacturer’s instructions. Afterward, primers and probes complementary to specific sequences within the lentiviral vector were used to amplify DNA. The standard curve was established using serial dilutions of the plasmid encoding the transgene. Amplifications were monitored using the ABI 7500 Real-Time PCR System (Applied Biosystems, Thermo Fisher Scientific, USA) according to the manufacturer’s instructions^2^.

**Statistical analysis.**

Statistical analyses were performed using SPSS 25.0 (SPSS Inc., Chicago, IL, USA). Categorical variables are expressed as percentages. The Kaplan‒Meier method was used to estimate the survival rate. The log-rank method was used to compare differences. Tables and graphs were designed using PowerPoint (Microsoft, Redmond, WA, USA) and Prism 8 (GraphPad Software Inc.). *P* values <0.05 were considered statistically significant.

**Overall survival and leukemia-free survival analysis.**

Subgroup analysis was used to analyze the factors affecting the prognosis of patients. Age, white blood cell (WBC) count at initial diagnosis and cytogenetic risk group were incorporated. Patient age >35 years old was shown to be associated with OS outcomes (*p* = 0.014, HR = 0.018) without a significant difference in LFS outcomes (*p* = 0.442, HR = 0.354) (Supplementary Fig. 3B). In the subgroup analysis of WBC counts and cytogenetic risk, patients with a WBC count above 30×10^9^/L had a trend of poor prognosis. However, no difference was found in OS and LFS outcomes (*p* = 0.414 and *p* = 0.327). Cytogenetic risk had no effect on patients’ LFS and OS outcomes (*p* = 0.854 and *p* = 0.763) (Supplementary Fig. 3C, D).

Guidelines and previous studies have demonstrated that age >35 years old, leukocyte count >30×10^9^/L at initial diagnosis, and poor genetic risk are risk factors affecting the prognosis of B-ALL patients^3^. In our research, we found that age over 35 years could affect patient OS outcomes (*p* = 0.014), but no difference was found in LFS rates (*p* = 0.442). Patients with leukocyte counts greater than 30 ×10^9^/L at the initial diagnosis risk appeared to exhibit worse OS and LFS outcomes, but there was no statistically significant difference. Genetic risk had no effect on patients’ LFS and OS outcomes (*p* = 0.854 and *p* = 0.763). However, the small sample size may have affected the preliminary results, and we will expand the sample size to verify the results in the future.

**References**

1. NCCN Clinical Practice Guidelines in Oncology — Management of Immunotherapy-Related Toxicities (2022 Version 1.0).

2. Kang L, Tang X, Zhang J, Li M, Xu N, Qi W, et al. Interleukin-6-knockdown of chimeric antigen receptor-modified T cells significantly reduces IL-6 release from monocytes. Exp Hematol Oncol. 2020;9:11.

3. Wolach O, Amitai I, DeAngelo DJ. Current challenges and opportunities in treating adult patients with Philadelphia-negative acute lymphoblastic leukaemia. Br J Haematol. 2017;179(5):705-723.

**Tables and figures**

**Supplementary Table 1. Baseline characteristics of patients.**

|  | **Total** |
| --- | --- |
| Number of patients, n | 12 |
| **Sex, n (%)** | |
| Male | 7(58.3) |
| Female | 5(41.7) |
| **Age, n (%)** | |
| 15-35 | 9(75) |
| >35 | 3(25) |
| **Diagnosis, n (%)** | |
| Ph^-^B-ALL | 9(75) |
| Ph^+^B-ALL | 2(16.7) |
| Ph-like B-ALL | 1(8.3) |
| **Cytogenetic risk group*, n (%)** | |
| Poor | 7(58.3) |
| Good | 5(41.7) |

Abbreviations: Ph^-^B-ALL = Ph-negative B-lymphocytic leukemia. Ph^+^B-ALL = Ph-positive B-lymphocytic leukemia. Ph-like B-ALL = Ph-like B-lymphocytic leukemia.

*: Genetic risk stratification was cited in the NCCN guidelines (version 1.2021).

**Supplementary Fig. 1. Clinical trial procedure.**


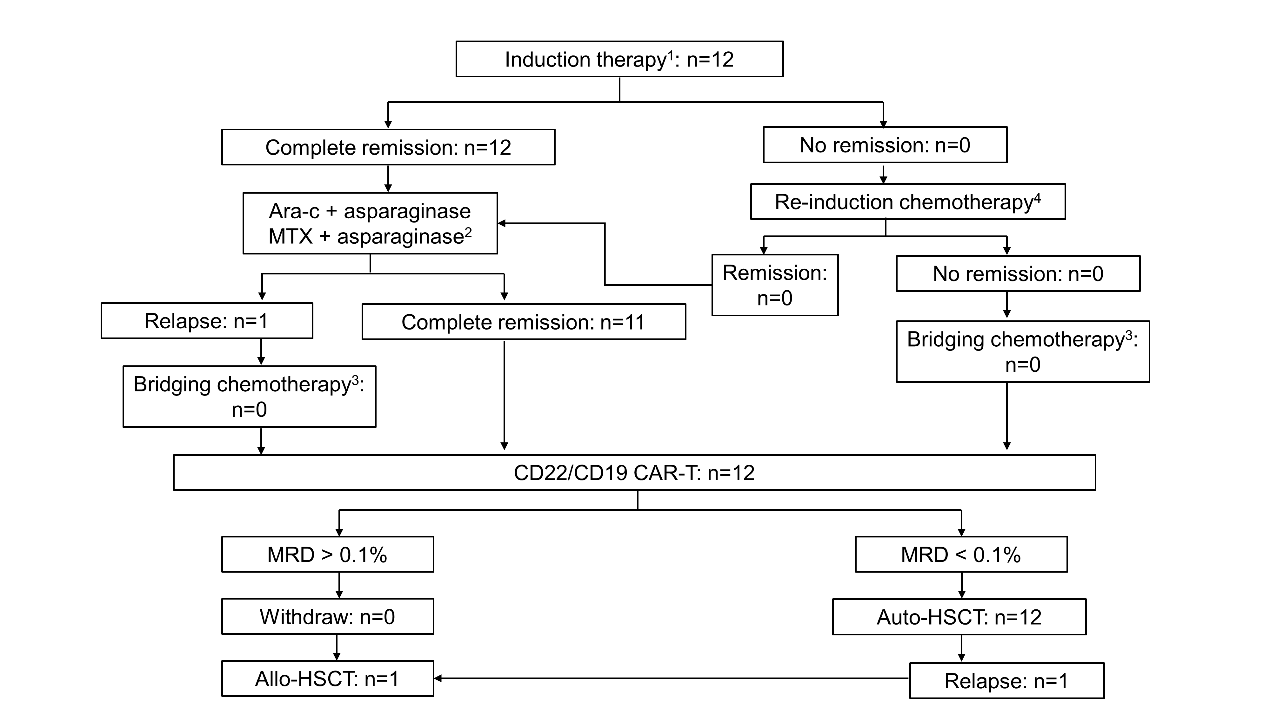


^1^The IVP regimen was used as an induction chemotherapy as previously described in the method. ^2^Patients received high dose cytarabine + asparaginase and methotrexate + asparaginase regimens as consolidation chemotherapies if they were in complete remission. ^3^Patients who experienced relapse after consolidation chemotherapy underwent bridging therapy before CD22/CD19 CAR-T therapy or received CD22/CD19 CAR-T therapy directly. This protocol did not allow other therapies related to CD22 or CD19 targets. ^4^Patients who received reinduction chemotherapy could choose the original regimen or other regimens depending on tumor burden and tolerability.

**Supplementary Fig. 2. CAR-T-cell infusion.**


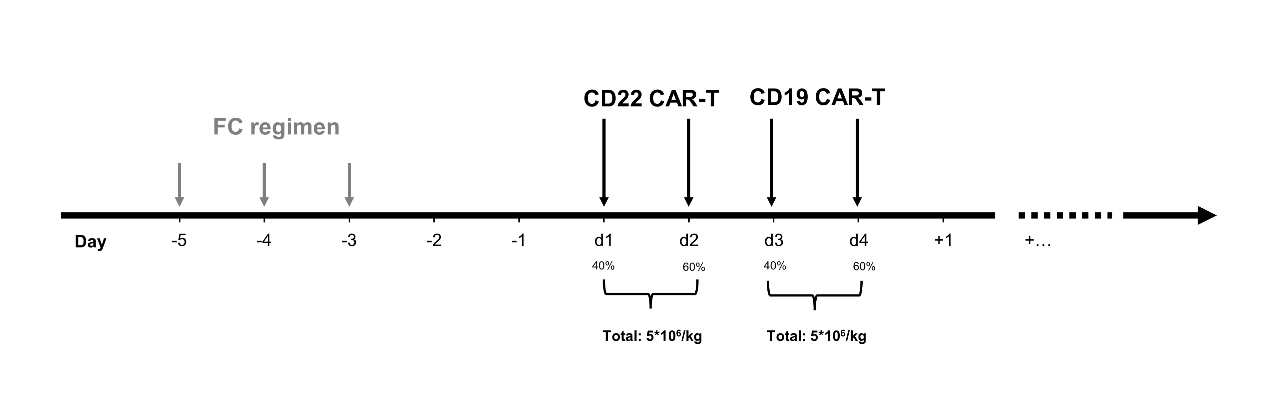


Lymphodepleting chemotherapy, including fludarabine and cyclophosphamide, was performed 5 days prior to CD22/CD19 CAR-T infusion. A separate dose of 5×106/kg CD22 CAR-T and CD19 CAR-T cells was sequentially infused within four days.

**Supplementary Fig. 3. Overall survival and leukemia-free survival analysis.**


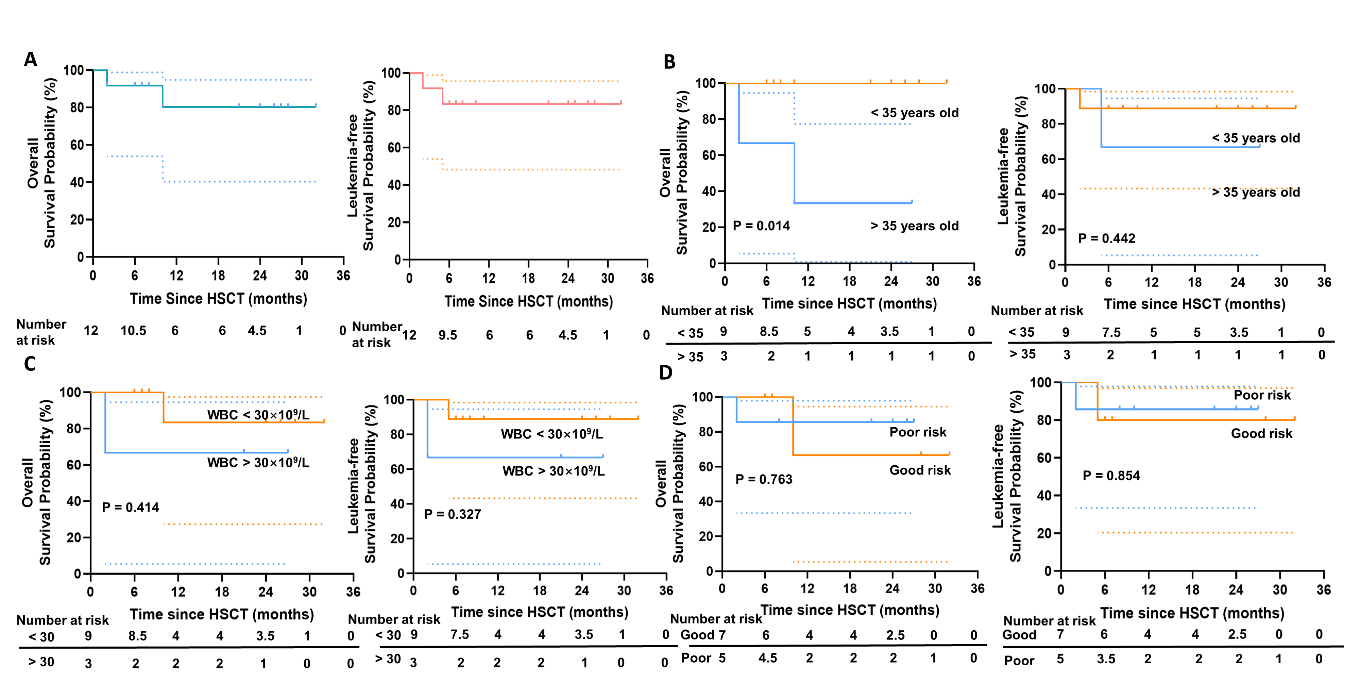


(A) The Kaplan‒Meier method was used to estimate the OS and LFS outcomes of all the patients. (B-D) Patients were grouped according to age, WBC counts at initial diagnosis, and cytogenetic risk stratification. Log-rank analysis was used to compare the differences in LFS and OS outcomes between the groups. Patient age >35 years was associated with OS outcomes without a significant difference in LFS outcomes. The dashed lines in A–D denote the 95% confidence interval.
